# Food Limitation but Not Enhanced Rates of Ejaculate Production Imposes Reproductive and Survival Costs to Male Crickets

**DOI:** 10.3390/cells10061498

**Published:** 2021-06-15

**Authors:** Saoirse McMahon, Magdalena Matzke, Cristina Tuni

**Affiliations:** Department of Biology II, Ludwig Maximilians University of Munich, Grosshaderner Str. 2, 82152 Planegg-Martinsried, Germany; saoirse.mcmahon@campus.lmu.de (S.M.); matzke@biologie.uni-muenchen.de (M.M.)

**Keywords:** reproductive costs, trade-offs, Gryllid, condition dependence, sperm quality, ejaculates

## Abstract

Estimating costs of ejaculate production is challenging. Metabolic investment in ejaculates may come at the expense of other physiological functions and may negatively affect future reproduction and/or survival. These trade-offs are especially likely to occur under constrained resource pools (e.g., poor nutrition). Here, we investigated costs of ejaculate production via trade-offs in the field cricket *Gryllus bimaculatus*. We experimentally increased rates of ejaculate production, while keeping an unmanipulated group, in adult males kept at high and low feeding regimes and tested the effects of our treatments on (i) somatic maintenance (i.e., changes in male body mass), (ii) future reproduction (i.e., the likelihood of producing a spermatophore and the viability of its sperm), and (iii) lifetime survival and longevity. We predicted investment in ejaculates to impinge upon all measured responses, especially in low-fed individuals. Instead, we only found negative effects of food limitation, suggesting low or undetectable costs of spermatophore production. High mating rates may select for males to maximize their capacity of ejaculate production, making ejaculate traits less prone to trade-offs with other fitness-related life history traits. Nevertheless, males were impaired due to nutrient deficiency in producing viable ejaculates, suggesting condition-dependent costs for ejaculate production.

## 1. Introduction

The view that costs of gamete production are sustained exclusively by females has been largely challenged; females do incur relatively higher costs for producing a small number of large eggs (for example, in terms of the gamete biomass production rate [1]), yet costs for ejaculate production in males are not trivial [1,2,3,4,5]. Ejaculates consist of multiple components that function as a unit, including sperm as well as a number of proteins and peptides in the seminal fluids [6,7]. They are favored by natural selection to ensure male fertility and by post-mating sexual selection to maximize male siring success during competitive fertilizations in polyandrous systems [8,9]. Hence, males should generally be under selection to invest greatly in each of their ejaculates, increasing rates of sperm production [10,11] and possessing fast-swimming and viable sperm to increase sperm competitiveness and siring success [8]. However, quantifying the costs of ejaculate production remains challenging. A few studies have addressed the physiological costs of ejaculate production through estimates of the basal metabolic rate [12,13] and/or energy expenditure (i.e., reserves of glycogen, lipids, protein [14], caloric analyses [15]), while most failed to disentangle these costs from the costs of mating [16]. Trade-offs are generally indicators of costs, where the high energetic demand of reproduction is expected to negatively impact future reproduction and survival [17,18,19,20]. Our understanding of reproductive trade-offs is rooted in the idea that, given individuals’ limited resource budget, any investment in one function (e.g., sperm production) comes at the expense of investment in other functions (e.g., soma maintenance) [18,19]. Energetic investments in ejaculate production have, in fact, been reported to trade against a number of physiological functions. For example, males undergoing sperm production are known to quickly lose body mass [21,22], to suffer from weakened immunity [23] and reduced survival [5], and to lower their investments in other aspects of reproduction such as secondary sexual traits [24]. Intensified ejaculate production may also reduce males’ ability to produce viable ejaculates in subsequent mating events [25,26,27], as frequent mating can deplete sperm and seminal fluids, the latter being important in ensuring sperm survival [6,28]. Importantly, since the quantity of metabolic resources available for reproduction is largely determined by variation in nutrient intake [29], energetic limitations may mediate individual resource allocation trade-offs between ejaculate production and other functions or traits [30,31,32,33]. Not surprisingly, trade-offs are most likely to appear under constrained resource availability (e.g., poor feeding conditions) [34,35,36].

Insects are a particularly valuable taxonomic group for advancing our understanding of the costs of ejaculate production [37,38,39,40]. Here, we use the common two-spotted field cricket, *Gryllus bimaculatus,* to investigate the costs of ejaculate production on male investment into soma maintenance, future reproduction, and survival and ask whether such costs are mediated by variation in food availability. Ejaculates of field crickets are packed into discrete spermatophores (i.e., protein capsules filled with sperm and accessory fluids), which are transferred to females upon genital coupling during copulation [41,42]. Spermatophores are located in a pouch on the tip of the male’s abdomen and can be easily sampled from males without major disruptions and, most importantly, without the need for a mating interaction with a female [43]. This allows disentangling ejaculate production from the mating event. In our study, we experimentally increased rates of spermatophore production by repeatedly removing spermatophores from males—while keeping an unmanipulated control group—from individuals that were reared under either high or low feeding regimes as adults. We then measured (i) changes in male body mass, (ii) the likelihood of producing a spermatophore and its quality (i.e., sperm viability), and (iii) longevity and lifetime survival in males, in order to understand whether increased spermatophore production trades against investment in somatic maintenance, future fertilization, and lifespan, respectively. Sperm viability, defined as the proportion of live cells within an ejaculate, is a well-justified metric for ejaculate quality [44] and is the main predictor for the outcome of competitive fertilizations in insects [45,46]. Sperm viability is also known to co-vary with the intensity of post-mating selection, with polyandrous species possessing higher sperm viability than their monogamous relatives (see [47] for insects, [48] for mammals). This suggests that under an intense sperm demand due to sperm competition or enhanced mating rates to ensure female sperm supply, males may evolve mechanisms to preserve sperm viability, for example, through higher resistance to stressors that could impact sperm integrity and function [49]. This may apply to our study species, known to be polygynandrous [50]. On the other hand, increased metabolism needed to fulfil an intensified ejaculate demand could reduce the efficiency of sperm production and maturation (spermatogenesis), resulting in a higher occurrence of cell defects, as reported for DNA sperm damage in rodent species with the highest levels of sperm competition [51]. Sperm viability may therefore decrease significantly across consecutive mating events, resulting in a decline in male fertility (e.g., the cockroach *Nauphoeta cinerea* [52]). Food restrictions may further exacerbate the negative effects of enhanced ejaculate production on sperm viability as energetic restrictions may reduce the range of seminal fluid proteins synthesized [53].

By applying an experimental treatment that increases rates of spermatophore production, we hypothesized intensified ejaculate production to carry reproductive and survival costs for males. We expected these costs to be revealed in experimentally manipulated males through (i) a drop in male body mass, (ii) a lower likelihood of spermatophore production and lower sperm viability within the spermatophore, and (iii) reduced longevity and survival probabilities. These costs should be more pronounced in energetically constrained males from the low feeding treatment. We found, instead, that restricted feeding conditions, but not intensified spermatophore production, imposed reproductive and survival costs on males.

## 2. Materials and Methods

### 2.1. Animal Rearing

Crickets of the species *Gryllus bimaculatus* used in our study were part of a large, outbred laboratory population, originated from wild-caught animals (approximately 200) collected in Tuscany (Italy) during summer 2018. Crickets were kept in multiple tanks (20 × 37 × 30 cm) in a climate room at a constant humidity (65%) and temperature (28 °C), with a 14:10 h light/dark cycle at the Ludwig Maximilians University of Munich (Germany). Each tank hosted approximately 30–40 crickets and was equipped with an egg carton to provide shelter and ad libitum access to dry cat food (Ja! Knusper-Mix Rind & Gemüse), fish flakes (sera^®^ Pond flakes), and water (using water vials with cotton stoppers). Tanks were kept at equal sex ratios and provided with small cups (diameter × height: 7 × 4.5 cm^3^) with moist soil for females to lay their eggs upon reaching adulthood. The offspring were raised communally as described above. After three generations, randomly chosen females were mated either monogamously (one female mated to one male for 3 times; *n* = 120) or polyandrously (one female mated to three consecutive males, *n* = 80) as part of a separate experiment. Animals were paired inside open arenas (15 × 15 × 6 cm^3^), and mating events were observed. Once mated, females were placed in individual tanks (30 × 18 × 20 cm^3^) provided with two successive oviposition cups (one per week) that were collected after one week, and eggs were allowed to hatch. Nymphs were raised communally within their mating treatment background (monogamous and polyandrous), and at their penultimate nymph stages, randomly chosen males were isolated into containers (10 × 10 × 9 cm^3^) equipped with food, shelter, and water. They were checked daily for emergence to adulthood.

### 2.2. Experimental Treatments

At their final molt, animals were randomly allocated to different life-lasting food treatments. Food treatments consisted of males with high food availability (high-fed, HF, *n* = 54) receiving 0.015 g of fish flakes, and with low food availability (low-fed, LF, *n* = 54) receiving 0.003 g of fish flakes, every three days. Such feeding regimes were chosen based on their significant effect on the male body condition reported in the field cricket *Gryllus campestris* [54]. Twelve days after the start of the feeding regimes, animals from each food treatment were then further randomly assigned to each one of the two treatments: for 7 consecutive days, spermatophores were either experimentally removed from males twice per day (sprmt-removal) to enhance spermatophore production, or spermatophores were not removed (sprmt-control). This resulted in 4 different experimental groups, LF sprmt-control (*n* = 30), LF sprmt-removal (*n* = 24), HF sprmt-control (*n* = 27), and HF sprmt-removal (*n* = 27). Removal of spermatophores was conducted by gently pressing on the male genital opening and collecting the spermatophore with soft forceps. The release of a spermatophore generally triggers the production of a new spermatophore [55]. Following spermatophore discharge, a refractory period—in which *G. bimaculatus* males start to manufacture a new spermatophore—is known to occur, and in the presence of a female, such process may start after 5 min [56]. Studies have shown that it takes 70 min for complete formation of the spermatophore [41]. We conducted a supplementary study to assess the likelihood and timing of formation of a replacement spermatophore following experimental removal and show that in the absence of a female, approximately 50% of males produce a fully formed spermatophore within 120 min from removal (Appendix A). Hence, we removed spermatophores once in the morning and once in the afternoon with a 4-h interval to allow males ample time to replace the collected spermatophore. When removing spermatophores, given that not all males possessed one upon inspection, we recorded the daily number of harvested spermatophores across LF and HF males. During the 7 days of experimental spermatophore removal, the average daily number of spermatophores removed from each male (range 0–2) did not differ significantly between HF and LF males (mean ± SE, HF 1.02 ± 0.05, *n* = 27; LF 1.01 ± 0.06, *n* = 24; two-sample t-test: t = −0.49, df = 50, *p* = 0.63).

The control group was not handled intentionally to avoid any form of stress that could potentially lead to autonomous spermatophore extrusion. Spermatophore auto-expulsion, where males discard a spermatophore autonomously in the absence of mating (also known as “spontaneous cycle renewal”), is common among *G. bimaculatus* [42,57] and crickets in general, occurring at rates lower than the experimental treatment imposed in our study (i.e., in the absence of a female, 81–84% males produce one spermatophore per day in *Teleogryllus commodus* [58] and 87.5% once every 2.6 days for *Acheta domesticus* [59]). To validate these assumptions, we investigated auto-extrusion in a small number of *G. bimaculatus* males (*n* = 10) from our laboratory population. We inspected the genital opening of two-week-old adult males (raised as described above and isolated individually upon emergence to adulthood), and when a spermatophore was present, it was marked using either acrylic paint (IDENA) or a black permanent marker (edding^®^ 3000). We then inspected these males on the following day to assess whether males retained the marked spermatophore or not in their pouch for 24 h. We found that 3 males did not retain the marked spermatophore (1 male produced a new one, and 2 did not have a spermatophore), suggesting low rates of auto-expulsion in the absence of a female or mating event.

### 2.3. Body Mass Measures

Male body mass was measured using a KERN PKT (KERN & SOHN GmbH, Balingen, Germany) digital scale at three time points: (i) at adulthood before animals were randomly allocated to different life-lasting food treatments (measure 1), (ii) twelve days from the start of the feeding regimes before being randomly assigned to one of the two spermatophore removal treatments (measure 2), and (iii) at the end of the 7 days of spermatophore removal treatments (measure 3).

### 2.4. Spermatophore Production and Sperm Viability

On the day following the end of the spermatophore removal treatments, all individuals, from both treatments, were inspected for spermatophore production, and spermatophores were sampled for sperm viability assays following established procedures [60,61]. Spermatophore age was standardized by removing the spermatophore from all males on the day before the assay. Spermatophores were removed and placed into a 0.5 mL Eppendorf tube with Beadle saline (200 μL) for 10 min to allow sperm to exit the protein capsule. The naturally occurring evacuation tube was also removed to ease release of semen. A total of 5 μL of the sperm–saline solution was pipetted onto a microscopy glass slide and stained with the LIVE/DEAD^®^ sperm viability kit (Invitrogen, Molecular Probes Inc, Eugene, OR, USA). We used 5 μL SYBR (1:50) and 2 μL of propidium iodide (PI), incubating the sample for 5 min in darkness after each addition. A cover slip was added, and the solution was then viewed under a fluorescent microscope (Olympus BX61; Olympus, Tokyo, Japan) with live sperm displaying as green (due to SYBR) and dead sperm as red (due to PI). Live and dead cells were counted in a total amount of 300 cells. On five occasions, we did not reach a total count of 300 cells. We excluded 4 data points (1 LF sprmt-control, 1 LF sprmt-removal, and 2 HF sprmt-control) as the number of total cells present in the sample ranged between 14 and 62, suggesting potential methodological issues in sample collection.

### 2.5. Longevity and Lifetime Survival

At the end of the sperm assay, males were returned to their individual housing boxes and inspected every 3 days to score mortality, until no surviving males remained. Mortality rates were also noted during the food and spermatophore removal treatments.

### 2.6. Statistical Analysis

All analyses were conducted using R version 4.0.1 [62].

Body mass. A t-test was used to test differences in male body mass measured before random allocation to the high and low feeding regimes (measure 1) to ensure lack of initial bias. To analyze whether our experimental procedure (food and spermatophore removal treatments) affected the change in male body mass (difference between the body mass before and after the spermatophore removal treatment, i.e., measure 2–measure 3), we ran a generalized linear mixed model (GLMM) using food treatment (HF and LF), spermatophore removal treatment (sprmt-control and sprmt-removal), and their interaction, as well as the measure (before and after spermatophore removal), as fixed factors in the model. To account for repeated measures, an individual male ID was included as a random effect. F and *p*-values were obtained using the univariate Anova function (car package). Here, and below, we also included the mating background of the animals’ mothers (offspring of females mated polyandrously or monogamously) as a factor in the model to account for potential biases resulting from this approach (Table S1). If the term was nonsignificant, we removed it and compared the simplified model using the Akaike information criterion (AIC) [63] (Table S2).

Spermatophore production and sperm viability. To test the effects of food treatment (HF and LF), experimental spermatophore removal (sprmt-control and sprmt-removal), and their interaction on the likelihood of producing a spermatophore at the end of the experimental manipulation (proportion of males with a spermatophore), we ran a GLM using a binomial distribution (GLM-b) and a logit link function. We analyzed the proportion of live cells (number of live cells out of the total number of cells counted) in the spermatophore, using the same model structure but including an individual male ID as a random effect to account for overdispersion (therefore running a GLMM) [64].

Longevity and lifetime survival. We ran two GLMs to test the effects of food treatment (HF and LF), experimental spermatophore removal (sprmt-control and sprmt-removal), and their interaction on (i) the proportion of males that survived the experimental treatments (GLM-b), and (ii) male lifespan (i.e., the total number of days an individual survived, log-transformed). To analyze if lifetime survival probabilities were affected by our experimental treatments, we carried out a Kaplan–Meier survival analysis to create survival curves and tested significance using a multivariate cox regression analysis on our lifetime data using the *survminer* package. Time (in days) was defined as the response variable with food treatment (HF and LF), spermatophore manipulation (sprmt-control and sprmt-removal), and their interaction as the independent variables. This is a non-parametric test to estimate the probability of survival at any given time interval in the data. Mating background was included in all models.

## 3. Results

### 3.1. Male Body Mass

**Figure 1:**
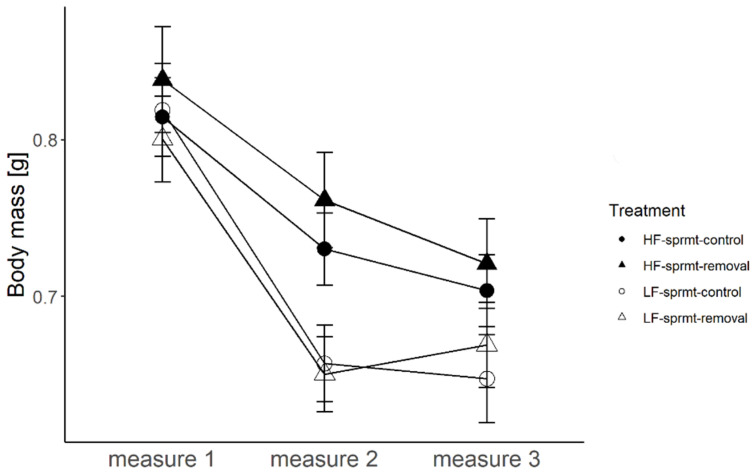
Change in body mass of males exposed to high and low feeding treatments (HF and LF), with and without experimental spermatophore removal (respectively, sprmt-removal and sprmt-control) measured at three time points (measure 1, before the start of the experimental food treatments, measure 2 before the start of the spermatophore removal treatment, measure 3 at the end of the spermatophore removal treatment) before and after the spermatophore removal treatment.

Males allocated to the two food treatments (HF and LF) did not differ in their mean body mass prior to the start of the feeding regimes (measure 1, t-test, t = 0.75, df = 106, *p* = 0.46; Figure 1). Hereafter, the changes in sample size are driven by male mortality (see below) and two missing data points (LF sprmt-control). After 12 days of differential feeding regimes, males from the HF treatment had a significantly higher body mass than those from the LF treatment (measure 2, Figure 1; Table 1). Once the spermatophore removal treatment started, male body mass generally decreased with time and was significantly affected by the feeding treatment, with HF males possessing a higher body mass than LF males, but not by the spermatophore removal treatment, nor their interaction (Figure 1; Table 1). Estimated effect sizes and 95% CIs around the mean of predictors are reported in Table S3.

### 3.2. Spermatophore Production and Sperm Viability

**Figure 2:**
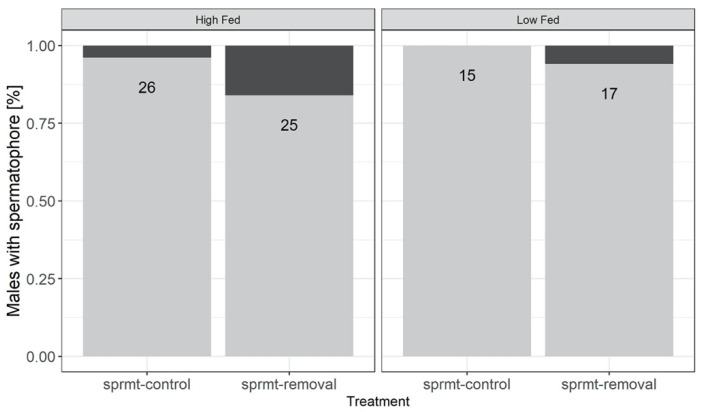
Proportion of males possessing a spermatophore at the end of the experimental treatment. Males were exposed to high and low feeding treatments (HF and LF), with and without experimental spermatophore removal (respectively, sprmt-removal and sprmt-control). Numbers inside bars are total sample sizes.

The likelihood that a male produced a spermatophore at the end of the experimental treatments was affected by the food treatment, with a higher proportion of LF males having a spermatophore compared to HF males (Figure 2; Table 1).

**Figure 3:**
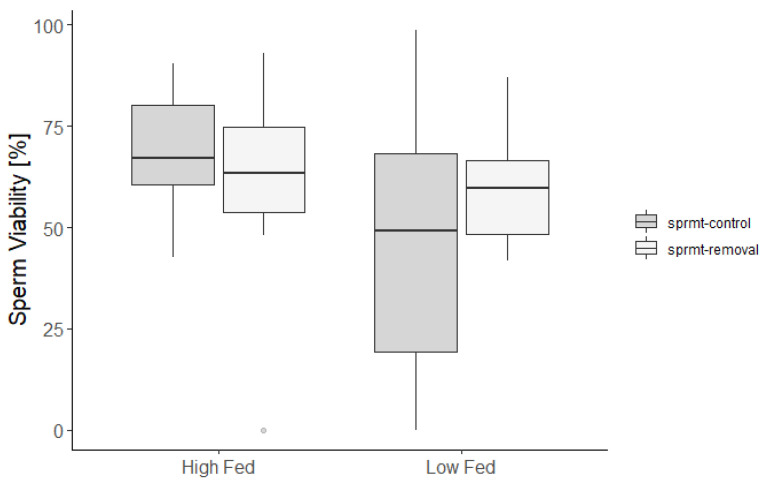
Proportion of live cells in the ejaculate of males exposed to high and low feeding treatments (high-fed and low-fed), with and without experimental spermatophore removal (respectively, sprmt-control and sprmt-removal).

Sperm viability was higher in HF males compared to LF males and was not affected by the spermatophore removal treatment (Figure 3; Table 1).

### 3.3. Longevity and Lifetime Survival

**Figure 4:**
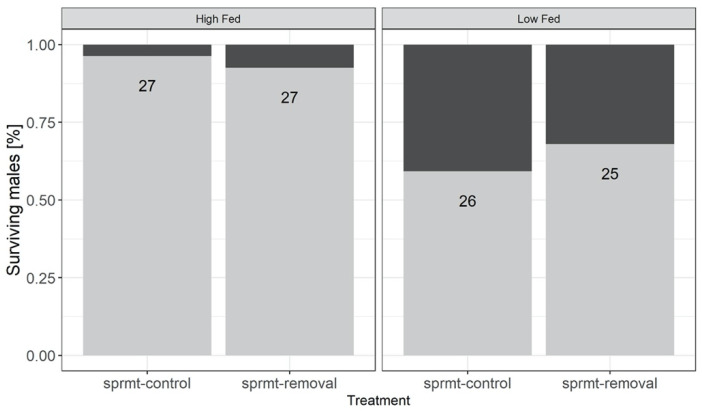
Proportion of males surviving the spermatophore removal treatment exposed to high and low feeding treatments (high-fed and low-fed), with and without experimental spermatophore removal (respectively, sprmt-control and sprmt-removal). Numbers inside bars are total sample sizes.

After 12 days of differential feeding regimes, two males from the LF treatment died. The proportion of males that survived the 7-day spermatophore removal treatment was significantly affected by the feeding treatment (94.4% HF and 59.3% LF) and by the spermatophore removal treatment (sprmt-control 77.4% and sprmt-removal 80.8%) (Figure 4; Table 1).

**Figure 5:**
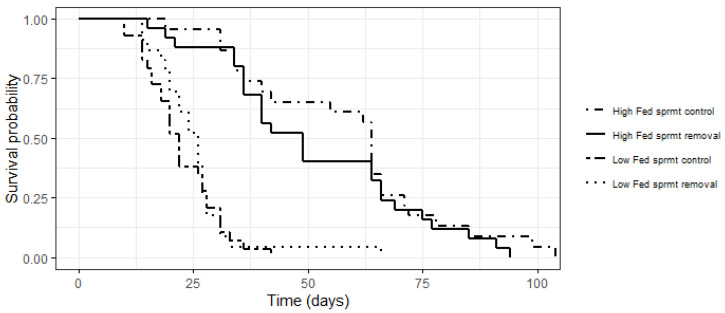
Lifetime survival probabilities of males exposed to high and low feeding treatments (high-fed and low-fed), with and without experimental spermatophore removal (respectively, sprmt-removal and sprmt-control).

Survival probabilities were significantly higher for HF males compared to LF males (HR = 9.77, df = 1, *p* < 0.0001), but did not differ between males with and without spermatophore removal (HR = 1.26, df = 1, *p* = 0.440), or in the interaction between food treatment and spermatophore removal treatment (HR = 0.59, df = 1, *p* = 0.203) (Figure 5).

Overall, we found a significant effect of the food treatment on male longevity (mean number of days alive (±) SE; HF 53.92 ± 3.16, *n* = 50; LF 24.12 ± 1.23, *n* = 54), while there was no effect of spermatophore removal (mean number of days alive (±) SE; sprmt-control 37.96 ± 3.21, *n* = 54; sprmt-removal 38.98 ± 3.02, *n* = 50) (Table 1).

## 4. Discussion

Organisms must allocate limited resources among competing life history functions and traits. When the reproductive effort increases, individuals’ feeding state may influence how the allocation of resources is partitioned [29], with allocation trade-offs being more pronounced in low-condition individuals. Despite the hypothesized costs associated with ejaculate production, our study did not unveil a direct physical trade-off between the energy allocated to enhanced rates of spermatophore production and that which is allocated to other organismal functions, such as somatic maintenance, future fertilization, and survival. This is in net contrast to studies reporting current vs. future reproductive trade-offs and/or reproductive vs. survival trade-offs in males [3,16,65,66]. 

We instead found that restricted feeding conditions imposed the highest costs on male crickets, leading to reduced body mass, lower viability of sperm, and impaired lifetime survival. The negative effects of low feeding regimes are not surprising, as resource availability plays a central role in individuals’ investment in life history traits, such as growth, survival, and reproduction [18,19,33]. The feeding regimes applied led to divergence in male body mass, with low-fed males losing more weight, especially in the first 12 days of differential food treatment. High-fed males had higher survival probabilities and lived twice as long as low-fed males. Interestingly, our results show condition-dependent differences in sperm viability, as the proportion of live sperm encapsulated in the spermatophore was, although not strongly, positively affected by access to food. The negative nutrient-dependent effects uncovered in our study suggest that dietary restrictions may strongly limit male mate acquisition and competitive fertilizations. Female crickets are indeed known to select mating partners based on variation in song parameters (i.e., higher chirp rates) which depend on male nutrient intake [67,68,69], and to prefer larger males [70], which are also more successful at defeating other males during aggressive agonistic interactions over the control of breeding territories [71]. Limited access to food may, however, also compromise male fertilization success when competing against ejaculates of rivals by enhancing sperm mortality [45]. Whereas it is well established that males acquiring more energetic resources are better at investing in costly secondary traits such as weapons (e.g., antlers, horns) or ornaments (e.g., long and colorful plumages) [72,73], condition dependence of ejaculates has been long debated. There is, in fact, contrasting evidence from empirical studies showing either positive [4,74,75], negative [76], or no [77] dietary effects on ejaculate traits. A recent meta-analysis showed that, despite the fact that the condition dependence of ejaculate traits is taxonomically widespread, traits differ in their response, with seminal fluids being strongly condition-dependent, while sperm traits are only moderately (i.e., sperm numbers) and less consistently reduced (i.e., sperm length, movement, viability) under nutrient limitation [53]. Studies on insects show consensus on the small or lack of effects of food availability and diet on sperm viability when testing for pollen restriction in the honey bee [77], protein restriction in male ants [78], protein and carbohydrate intake in the cockroach [79], poor nutrition in the leaf-footed cactus bug [80], or diet type in the milkweed bug [81]. Studies on field crickets have, instead, been suggestive of positive effects of body resources on sperm viability by reporting higher proportions of live sperm in the ejaculate for heavier males (*Gryllus bimaculatus* [60]). Apart from our study which establishes a causal relationship through experimental manipulation of food availability, manipulation of macro- and micronutrients is also known to affect trait expression in the species *Teleogryllus oceanicus* [82], with males producing more viable sperm under higher consumption of micronutrients, but the lowest amounts on high-protein diets.

A possible explanation for the apparent low or negligible costs of enhanced spermatophore production reported in our study may reside in the fact that high mating rates, known to occur in natural populations of *G. bimaculatus* [50], may select for males to maximize fertilization rates through an increased capacity of spermatophore production [83]. *G. bimaculatus* males are also known to mate repeatedly throughout their adult life [84], further suggesting they can bear an elevated lifetime reproductive potential. Our findings may also point to potentially low energetic demands of multiple-spermatophore manufacturing. Interestingly, to this end, we found that despite the decreased body mass, low-fed males were able to maintain rates of daily spermatophore production similar to those of well-fed males, and, at the end of the experimental manipulation, the likelihood of low-fed males possessing a spermatophore was even higher than for high-fed males. We also show from an auxiliary study conducted without diet manipulation that investigates the timing and likelihood of spermatophore formation that male body mass does not positively correlate with the probability of producing a replacement spermatophore after discharge. This finding is also known for other cricket species, such as *Gryllus veletis* and *Gryllus pensilvanicus* [85], and suggests a lack of energetic limitations for spermatophore production. Spermatophores of field crickets are relatively small [86], hardened, sack-like, sperm-containing ampullae. In *Gryllus bimaculatus*, they constitute 0.18% of the male’s body weight [84]. Males can initiate spermatophore production 5 min after discharge [41] and complete its syntheses within 1 h (our study and [41]). In many other species of Orthopterans, spermatophores instead include the spermatophylax, a large gelatinous non-sperm component rich in proteins [87,88] which surrounds the ampulla, and that is eaten by the female at mating. These spermatophores may represent a large percentage of the male body mass with reports of up to 26% of the male’s weight in certain bushcrickets (i.e., genus *Poecilimon* [89,90]). Despite being rare in field crickets, the spermatophylax is present in the decorated cricket *Gryllodes sigillatus*, where costs of production are supported by long refractory periods (i.e., it takes 3 h to synthesize a new spermatophore [91]) and by trade-offs between increasing rates of spermatophore production and immunity, revealed by applying a very similar manipulation to that of our study (i.e., 5 consecutive days of spermatophore removal) [38].

Considering spermatophore production, an entirely cost-free physiological process may, however, be unlikely. We show that despite the fact that nutritionally restricted males can afford to produce spermatophores at high rates, they are impaired in producing high-quality ejaculates, suggesting that, to some extent, certain aspects of ejaculate production are costly. It is plausible that sperm viability is modulated by seminal fluid proteins that serve to nourish sperm cells [6,49,92], with seminal fluid production itself being largely affected by diet [93]. We cannot exclude that a more stringent experimental treatment (i.e., higher rates of spermatophore removal) could have revealed measurable costs in our target traits and functions, or that, alternatively, trade-offs occur between functions other than those addressed in our study. For example, if males hold their investment constant by maintaining high spermatophore production rates, they may need longer refractory periods between mating events that may, overall, lead to reduced lifetime reproductive success [84]. How quickly males are able to produce sperm and replenish sperm reserves strongly affects their fertilization advantage [94], as possessing a ready-formed spermatophore would allow promptly courting females upon an encounter and, if accepted, readily transferring sperm. Males investing in spermatophore production may otherwise reduce their investment in other fundamental and costly secondary sexual traits, such as fighting [24]. Male *G. bimaculatus* that win fights against rivals are shown to produce lower-quality ejaculates (less viable sperm), suggesting a trade-off in pre-mating and post-mating competitiveness [60]. Unknowingly, sperm characteristics other than the one measured may have been impaired by our experimental treatment. The most common negative effect of repeated mating events across a wide range of species is sperm depletion [95,96,97]. In field crickets, the number of sperm encapsulated in the spermatophore during the second and third mating events contains 50–60% of the sperm transferred during the first mating event [98]. A decline in ejaculate mass with an increasing number of mating events is also documented in various species of seed beetles [25] and in lepidopterans, where spermatophores, following a previous mating experience, are smaller [26]. Over consecutive mating events, males may also become depleted of other important components of their ejaculates. For example, with repeated mating events, male *Drosophila melanogaster* recover their ability to manufacture and transfer seminal fluid proteins only after 3 days of sexual inactivity [99]. Male accessory glands, responsible for secretion of seminal fluids, are known to reduce in size after mating in several species [100,101]. Finally, sperm traits are also known to correlate negatively with each other [102], potentially masking the occurrence of trade-offs if only one trait is measured. Our findings may also stem from methodological differences with other studies addressing costs of reproduction for males. In order to exclusively target ejaculate production costs, we adopted a design that excludes mating. On the contrary, many of the studies reporting a decline in ejaculate quality, growth, and survival involved a female presence and/or allowed mating to take place [3,16]. These studies may not be able to fully distinguish between the effects of behavioral exhaustion derived from performing energetically demanding courtship [103] and/or those of copulation [104] from ejaculate production alone [5] in limiting the male fertilization potential. In addition, males may face strategic allocation decisions in the presence of varying mating opportunities, investing in each ejaculate in a way that maximizes their fitness return [105]. Males may, for example, partition their resource investment among multiple mating events, reducing sperm allocation per mating event [106,107], hence hindering interpretations of the exact constraints of ejaculate production.

Interestingly, crickets that were challenged by reduced access to food were able to maintain higher sperm viability under intensified spermatophore production. We here interpret these findings with caution. On the one hand, these may indicate that under harsh environmental conditions, such as nutrient restrictions, males that are exposed to enhanced reproductive effort may invest more in reproduction (e.g., keeping vital sperm cells). If an individual’s perception of the increased mortality risk increases, evolutionary theory indeed predicts an increased investment in current reproduction (namely, the terminal investment hypothesis) [34,108,109]. It is, however, also possible that if males in low feeding regimes were energetically impaired in their rates of spermatophore discharge (auto-extrusion) [110], they may have spent longer periods without active sperm production. Ejaculate quality after periods of abstinence from mating is known to only increase in subsequent mating events or ejaculations [111]. This is most likely due to sperm storage mechanisms and sperm aging lowering sperm performance, as sperm stored by males before mating may incur post-meiotic sperm senescence, leading to a decline in the number of viable sperm, and sperm motility and velocity [112,113]. In our study, we aimed to experimentally control differences in sperm age by removing the spermatophore from all males on the day prior to the sperm assays. However, older sperm may remain in the male reproductive tract prior to being loaded in the newly formed spermatophore [59]. Hence, increased rates of spermatophore production may have proved beneficial in maintaining viable sperm cells.

## 5. Conclusions

In conclusion, dietary restrictions may strongly limit male reproductive success through profound negative effects on important physiological functions (e.g., soma maintenance, sperm production, and survival), stressing the importance of individual resource availability. Yet, investment towards ejaculate production may not necessarily occur at the expense of such functions, even when resources are scarce [29]. Our findings suggest that ejaculate traits may be less prone to trade-offs with other fitness-related life history traits while unveiling condition-dependent costs. Indeed, although male field crickets appeared to bear the costs of producing multiple spermatophores, they were impaired from nutrient deficiency in producing high-quality ejaculates. This extends our understanding of the condition dependence of ejaculate quality, as a direct relationship between energy intake and sperm viability is seldom reported in insects.

## Figures and Tables

**Table 1:** Results of statistical models (GLMs unless specified) showing the effect of food treatment (high-fed and low-fed), experimental spermatophore removal (removal and control), their interaction, and the time points for body mass measures (measures 2 and 3) on male responses indicating investment in (i) soma maintenance (change in body mass), (ii) future reproduction (spermatophore production and sperm viability), and (iii) survival (% males surviving at the end of the experimental treatment) and longevity (number of days alive). Significant effects are shown in italics.

Response Variable		Effect (Wald X^2^ or F; df; *P*)			
	N	Food Treatment	Spermatophore Removal Treatment	Food x Spermatophore Treatment	Timepoint of Measure
Body mass ^1^	188	18.78; 1; <0.0001	0.0006; 1; 0.98	0.0001; 1; 0.99	41.53; 1; <0.0001
Spermatophore production (% males) ^2^	84	20.6; 1; <0.0001	0.72; 1; 0.4	1.12; 1; 0.29	-
Sperm viability (% live sperm) ^1,2^	73	3.97; 1; 0.046	0.08; 1; 0.77	3.53; 1; 0.06	-
Survival post-spermatophore removal (% males) ^2^	109	3.99; 1; 0.046	3.87; 1; 0.049	0.0; 1; 1	-
Longevity (N days alive)	104	104.2; 1; <0.0001	0.0001; 1; 0.99	3.68; 1; 0.06	-

^1^ GLMM. ^2^ binomial.

## Data Availability

Data are contained within Table S4 of Supplementary Material.

## Supplementary Materials

The following are available online at https://www.mdpi.com/article/10.3390/cells10061498/s1, Table S1: Results of statistical models including male family background (polyandrous or monogamous mothers). Table S2: Akaike information criterion value (AIC) for models including or not including male family background (polyandrous or monogamous mothers). Table S3: Estimated effect sizes and 95% CIs around the mean of predictors of body mass measured at two time points (measures 2 and 3), before and after the spermatophore removal treatment, including (+) or not including (-) male family background (polyandrous or monogamous mothers). Table S4: Full dataset.

## Appendix A

A total number of 38 males were used to assess the likelihood and timing of production of a replacement spermatophore following experimental removal, in the absence of a female. Animals belonged to a large outbred population originated from wild-caught individuals collected in Tuscany, Italy, during summer 2015. They were raised in the laboratory following standardized conditions, as described above. Males were isolated individually during their penultimate instar and used 3–4 weeks after adult eclosion. On the day of the test, after measuring male body mass using a KERN PKT (KERN & SOHN GmbH, Balingen, Germany) digital scale, the male’s spermatophore was experimentally removed with soft forceps. The male was then returned to its housing container, and his genital opening was inspected regularly after 10, 20, 30, 40, 50, 60, and 120 min. On the following day (24 h later), we inspected males that did not produce a spermatophore 120 min from removal. During each inspection, we noted whether males initiated spermatophore production. The process is visible, as the genital pouch at first contains white and soft material (at 10 and 20 min) to then become clear (at 30 and 40 min) and finally hardens (at 50 and 60 min) into a fully formed spermatophore. This process was observable in 47% of the males (*n* = 18), which possessed a fully formed spermatophore in their pouch 60 min from removal. Of the remaining 53% (*n* = 20) that did not initiate spermatophore production within the first 10 min, five had spermatophores after 120 min and the remaining never did on that day. However, they all possessed a spermatophore on the following day. We ran GLM binomial testing for the effect of male age and body mass on the likelihood of producing a replacement spermatophore within 120 min from removal. Interestingly, we found no effect of body mass on the likelihood to produce a replacement spermatophore, but older males were more likely to produce one (GLM-b, male age χ^2^ = 7.21, df = 1, *p* = 0.0073*; male body mass χ^2^ = 0.68, df = 1, *p* = 0.41). These results suggest an increase in reproductive investment with a decreasing reproductive value [34].
