# Supplementary material for: Food Limitation but Not Enhanced Rates of Ejaculate Production Imposes Reproductive and Survival Costs to Male Crickets
Source: Cells. 2021 Jun 15;10(6):1498. doi: 10.3390/cells10061498 (PMC8232169; doi:10.3390/cells10061498)
Supplement: Supplementary file 1 [file cells-10-01498-s001.zip › McMahon et al. 2021 Supplementary 1-3_track changes.pdf]

cells MDPI

Article

Food limitation but not enhanced rates of ejaculate production impose reproductive and survival costs to male crickets

Saoirse McMahon [1], Magdalena Matzke [1] and Cristina Tuni [1,*]

Supplementary Materials

Table S1. Results of statistical models (GLMs unless specified) showing the effect of food treatment (high fed and low fed), experimental spermatophore removal (removal and control), their interaction, the time points for body mass measures and male family background (polyandrous or monogamous mothers) on male responses indicating investment in i) soma maintenance (change in body mass), ii) future reproduction (spermatophore production and sperm viability) and iii) survival (% males surviving at the end of the experimental treatment) and longevity (number of days alive). Significant effects are shown in italics.

Response variable	Effect (Wald X^2 or F; df; P)					
	N	Food Treatment	Spermatophore removal treatment	Food x Spermt	Family background	Measure (1 and 2)
Body mass[1]	188	*19.89; 1; <.0001*	0.002; 1; 0.96	0.15; 1; 0.7	*10.74; 1; 0.001*	*40.91; 1; <.0001*
Spermatophore production (% males)[2]	84	*20.52; 1; <.0001*	0.65; 1; 0.42	0.99; 1; 0.32	0.26; 1; 0.61	-
Sperm viability (% live sperm)[1,2]	73	3.68; 1; 0.055	0.09; 1; 0.76	*4.23; 1; 0.04*	1.81; 1; 0.18	-
Survival (% males)[1]	109	*4.14; 1; 0.042*	*4.0; 1; 0.045*	0.0; 1; 1	0.24; 1; 0.63	-
Longevity (N days alive)	104	*103.1; 1; <.0001*	0.0001; 1; 0.99	0.0007; 1; 0.98	3.6; 1; 0.06	-

[1] GLMM

[2] binomial

Table S2. Akaike Information Criterion value (AIC) for models including (+) or not (-) male family background (polyandrous or monogamous mothers).

Models	+ Family background	- Family background
Body mass[1]	-397.4	-389.2
Spermatophore production (% males)[2]	104.1	102.3
Sperm viability (% live sperm)[1,2]	834.3	834.1
Survival (% males)[1]	28.8	27.1
Longevity (N days alive)	110.3	108.3

[1] GLMM

[2] binomial

Table S3. Estimated effect sizes and 95% CI around the mean of predictors of body mass measured at two time points (1 and 2), respectively before and after the spermatophore removal treatment, including (+) or not (-) male family background (polyandrous or monogamous mothers).

**Fixed effects**	**+ Family background β (95% CI)**	**- Family background β (95% CI)**
Intercept	0.71 (0.68, 0.75)	0.75 (0.71, 0.78)
Food Treatment (LF)	-0.09 (-0.13, -0.05)	-0.09 (-0.13, -0.05)
Spermatophore removal treatment (removal)	-0.006 (-0.05, 0.03)	-0.0005 (-0.04, 0.04)
Measure (Time point 2)	-0.04 (-0.044, -0.026	-0.04 (-0.04, -0.03)
Food LF x Spermt removal	0.015 (-0.05, 0.08)	0.0003 (-0.07, 0.06)
Mating background (PP)	0.079 (0.04, 0.12)	
**Random effects**	**σ^2 (95% CI)**	**σ^2 (95% CI)**
Individual	0.014 (0.013, 0.016)	0.016 (0.015, 0.017)
Residual	0.001 (0.0011, 0.0015)	0.001 (0.0011, 0.0015)
